# Differences in Phytobenthic Diatom Community between Natural and Channelized River Sections

**DOI:** 10.3390/plants12112191

**Published:** 2023-05-31

**Authors:** Igor Zelnik, Mateja Germ, Aleksandra Golob, Aleksandra Krivograd Klemenčič

**Affiliations:** 1 Department of Biology, Biotechnical Faculty, University of Ljubljana, Jamnikarjeva 101, 1000 Ljubljana, Slovenia; aleksandra.golob@bf.uni-lj.si; 2 Slovenian Environment Agency, Vojkova 1b, 1000 Ljubljana, Slovenia; aleksandra.krivograd-klemencic@gov.si

**Keywords:** diatoms, phytobenthos, channelized rivers, Slovenia, ecological types, diversity

## Abstract

The structure of phytobenthic diatom communities was studied to reveal differences between natural and channelized river sections in Slovenia. As part of the national monitoring of surface waters, samples of phytobenthos were collected at 85 sites throughout the country according to standard protocols. At the same time, basic environmental parameters were also assessed. Trophic (TI) and saprobic (SI) indices were calculated based on diatoms and other algae, while diversity indices and gradient analyses were performed only for the diatom community. The results showed that channelized rivers harbor significantly more diverse benthic diatom communities than natural sections, mainly due to the significantly higher number of motile diatom taxa that are able to take advantage of more nutrient-rich and less-shaded river sections because of their high adaptability. Selected environmental parameters explained 34% of the variability in diatom community structure when taxa were classified into ecological types. The removal of *Achnanthidium minutissimum* yielded clearer results (24.1%) than the total species matrix (22.6%). Therefore, we suggest excluding this taxon from calculations of TI, SI, or other indices when it is determined as *A. minutissimum* complex, because *A. minutissimum* complex was most abundant in both types of reaches in our study and has a wide ecological amplitude, which reduces the indicative power of the diatom community in the evaluation of environmental conditions and ecological status.

## 1. Introduction

**Figure 1:**
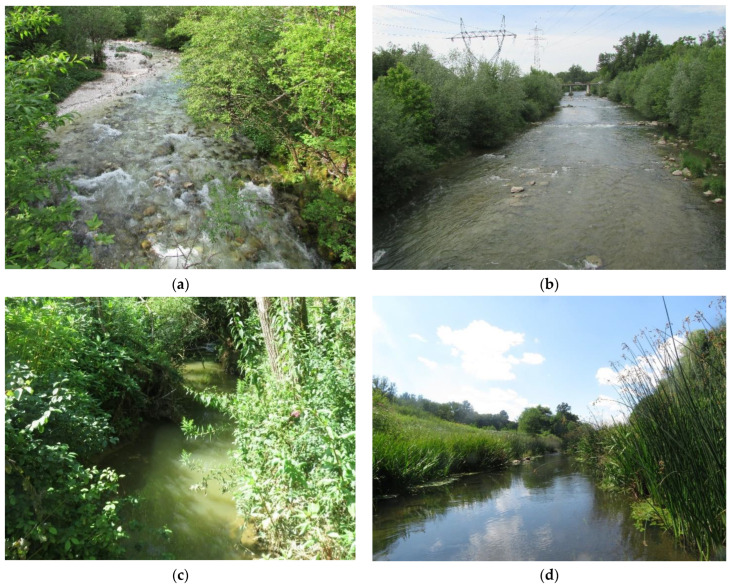
Both types of sections of the River Kamniška Bistrica—natural (**a**) and channelized (**b**) in the Alpine region (central Slovenia) and of the River Ledava in the Pannonian region—natural (**c**) and channelized (**d**) on the border with Hungary.

Hydromorphological alterations are among the worst anthropogenic pressures affecting European rivers and streams [1] and are often a reason for the poor ecological status of rivers [2]. Land use changes in river floodplains due to the expansion of settlements and agricultural land have led to the regulation of numerous rivers throughout Europe in the 20th century. It was believed that the regulations would reduce the flood risk to settlements in the former floodplains. According to Feld and Hering [3], the term hydromorphological degradation describes numerous impacts acting at different spatial scales. Erba et al. [4] divided hydromorphological alterations into three types: (a) channel resectioning or channelization, (b) impoundments, and (c) fine sediment accumulation with different effects. The channelization of rivers leads to a reduction in original habitat complexity and habitat availability in increasingly uniform riverbeds (Figure 1), e.g., Lau et al. [5]. A large number of freshwater researchers agree that regulated rivers represent degraded fluvial ecosystems, reflected in lower habitat and microhabitat diversity and consequently in lower species diversity [4,6,7,8].

Several studies have reported the degradation of biotic communities and lower species numbers in channelized river reaches compared to natural reaches for a variety of aquatic communities, such as fish [9] and benthic invertebrates [4,10,11]. However, the responses of aquatic communities to morphological alterations are not always obvious or unambiguous. Gebler and Szoszkiewicz [12] reported that the relative abundance and diversity of macrophytes mostly decreased with hydromorphological degradation. However, some sites with fewer changes allowed for the development of diverse macrophyte communities. Wyžga et al. [10] found that in rivers with short regulated sections, the number of fish species was not lower than in natural rivers. Moreover, the response of the benthic invertebrate community to channelization has been described as weak or unclear in many studies [13,14,15].

Straightened and constricted river channels have higher flow velocities, resulting in differences in species composition and their proportions in aquatic communities compared to intact sites [16]. Rheophilic fish and benthic invertebrate species replace those that prefer moderate or lower flow velocities; furthermore, the riverine communities of regulated river channels with higher flow velocities generally shift toward upstream riverine communities [17]. Szczepocka et al. [18] claim that for diatom communities as well, such pseudo-mountain conditions favor the establishment of taxa that prefer cold water with high O_2_ concentrations.

**Figure 2:**
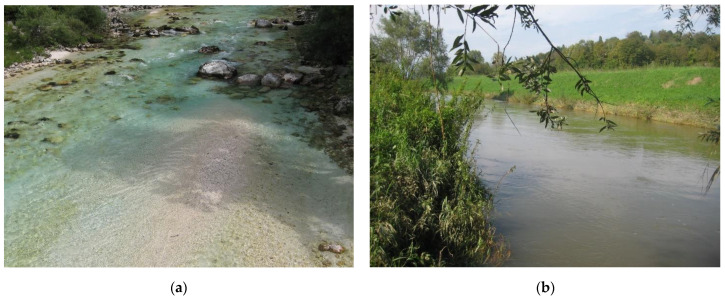
Natural section of the River Soča in the Alpine region, shaded by tall riparian vegetation (**a**) and a channelized section of the River Sotla (**b**) in the Pannonian region, where the riparian vegetation was removed on the left bank, which is the border with Croatia.

Another consequence of the channelization of rivers is their reduced ability to deal with pollutants in the form of degradable organic matter and inorganic nutrients [19]. Channelized rivers have lower efficiency of self-purification processes due to the lower specific surface area of the substrata that host biofilms (Figure 2), which include the major groups of organisms involved in the removal of dissolved organic matter and inorganic nutrients from water, as well as due to the shorter residence time of water in a given section of the river. Lynch et al. 2019 [20] reported that the physical heterogeneity of the river channel critically controls the residence time and concentration of dissolved organic matter (DOM) in the river. Periphyton, a community of microscopic algae, cyanobacteria, and heterotrophic microorganisms growing on the surface of various submerged substrata [21], and phytobenthos, a subcommunity that includes only primary producers according to the Water Framework Directive (WFD) [22], are the basis for self-purification processes within the riverine ecosystem because they have great potential for removing dissolved pollutants from the water [23]. Higher current velocities in straightened river channels generate more frequent and intense disturbances than in unregulated watercourses, which is reflected in lower species richness [24] and lower phytobenthic biomass. Lower phytobenthic biomass affects species at higher trophic levels, as algae and cyanobacteria are the principal primary producers in watercourses [25] and support various groups of invertebrates and vertebrates [26]. Due to its rapid response to environmental changes, phytobenthos is also a useful indicator of stream water quality [27].

An assessment of the ecological status of the surface waterbodies in the member states of the European Union (EU) also includes an evaluation of hydromorphology (hydromorphological quality elements). The biological quality elements (BQEs), which are considered to be the most sensitive to morphological alterations in rivers according to the WFD [22], are benthic invertebrates and fish that have been used for this part of the evaluation, while other BQEs, such as phytobenthos and macrophytes, have not been used in the mentioned metric. In Slovenia, the benthic diatom community is used as a proxy for the identification of eutrophication (trophic index (TI)) and organic pollution (saprobic index (SI)) in rivers as part of the surface water ecological status assessment of the BQE phytobenthos and macrophytes, according to WFD [22], since TI and SI enable the detection of nutrient and organic pollution, respectively [28]. Several other quality indices, such as the Specific Pollution Sensitivity Index (IPS), are also based on diatom data only [28,29]; however, they are used in other EU member states (e.g., France).

There is an approach that uses the functional characteristics of diatoms as an alternative to the taxonomic composition of diatoms for assessing the quality of aquatic ecosystems [28]. The classification of diatom taxa into ecological groups based on the different adaptive strategies proposed by Rimet and Bouchez [30] has proven to be very thorough and applicable. According to Rimet and Bouchez [30], diatoms are classified into four ecological types, namely low profile, high profile, motile, and planktonic, that have different adaptation strategies to, for example, disturbances by high current velocities and nutrient levels. Such a classification can strengthen relationships to specific environmental stressors or factors compared to species data [30]. Planktonic taxa (PL) are adapted to lentic ecosystems to slow their sedimentation and generally have thinner frustules than benthic species. The low-profile (LP) group includes taxa that are attached to the substrate with a valve face or at the pole and slow-moving taxa. They are more resistant to physical disturbances than others but do not prefer high nutrient levels [31]. The high-profile (HP) group includes taxa with larger cells, stalked species, or species that form different types of colonies. The taxa in this group are sensitive to physical disturbances.

The motile (M) taxa group includes fast-moving species that are capable of efficient locomotion and are adapted to disturbances by rapid water flow, as well as to high nutrient levels. Their ability to move efficiently enables them to select the most suitable microhabitat [32]. An approach based on functional classification is also useful in water quality assessment [33]. Despite the general opinion of limnologists about the low indicative value of benthic diatom communities for the assessment of hydromorphological pressures, there is a potential that the ecological types of diatoms reflect anthropogenic changes in river morphology.

The purpose of this study was to investigate how river channelization affects the diversity and structure of the benthic diatom community and whether diatoms also respond to this type of morphological alteration. Phytobenthic diatoms could be used as an additional BQE to complement the benthic invertebrates and fish that are used to assess hydromorphological pressures and whose validity is sometimes limited when assessing anthropogenic pressures on rivers in the form of hydromorphological alterations. The aims of this study were to find out (a) which environmental parameters significantly shape the diatom community composition and (b) whether there are differences in diatom community structure between natural and regulated rivers. The following hypotheses were made: (a) diatom species richness and diversity are higher in natural than in regulated waterbodies; (b) the values of TI and SI are significantly higher in regulated compared to natural rivers due to their lower self-purification capacity; and (c) different ecological diatom types respond differently to river channelization, indicating the possibility of using benthic diatoms for the assessment of anthropogenic morphological river alterations.

## 2. Results

### 2.1. Differences between Natural and Regulated River Sections

A total of 163 diatom taxa were identified in all 40 investigated rivers; most of them were at the species level, with some exceptions at the genus level. In natural river sections, 141 diatom taxa were identified in 65 samples (average 24.9), while in channelized river sections, 117 diatom taxa were identified in 20 samples, with an average number of 28.6 diatom taxa per sample (Table 1).

The proportions of ecological types of benthic diatoms differed between the natural and regulated river sections (Table 1), especially for the motile (M) taxa, which had a significantly (*p* = 0.024) higher proportion in regulated river sections. The difference in high-profile (HP) taxa between natural and regulated river sections was only marginally significant (*p* = 0.063), with a lower proportion in channelized river sections. There were also differences in the order of proportions of ecological diatom types between natural and regulated river sections; however, the low-profile type (LP) had the highest proportion in both groups. The second most common ecological type in the natural river sections was HP, while in regulated river sections, the M taxa had a higher proportion. The order of contribution to diatom diversity was also not the same for both river groups (Table 1). Species richness was highest among the M taxa in both groups, and the Shannon–Wiener diversity index (SDI) and diversity of the motile ecological type were significantly higher (*p* = 0.018) in regulated rivers. Although the LP taxa accounted for half of the valves found (57 and 50% for natural and regulated river sections, respectively), the SDI of the LP diatom metacommunity was lowest in both river groups, with significantly lower values (*p* = 0.026) for natural sections.

There were also significant differences in environmental parameters between the natural and regulated river section groups (Table 2), which could be the reason for differences in diatom community structure at the ecological type level (Table 3) and taxonomic composition (Table 4). There were highly significant differences (*p* < 0.01) in environmental parameters between the two groups for (a) altitude, as the channelized river sections were found at lower altitudes (*p* = 0.002), mostly in lowlands; (b) proportion of gravel (*p* = 0.003), which was higher in the natural river sections; and (c) conductivity, which was higher in regulated river sections (*p* = 0.005), reflecting a higher proportion of agricultural land adjacent to the regulated sections and their lower self-purification capacity.

### 2.2. Influence of Selected Environmental Factors on the Structure of the Diatom Community

The results of the direct gradient analyses provided a list of nine environmental parameters that significantly shaped the diatom community (Table 5), with conductivity and SI obtained on the basis of other algae, explaining the largest proportion of the variability (4.8 and 3.0). Overall, the explained variability of the diatom community was rather low. Replacing the taxa matrix with the matrix of ecological types resulted in a higher proportion of the explained variability of the diatom community by the same matrix of environmental parameters (34.4% instead of 22.6%, respectively). The set of statistically significant environmental variables that shaped the diatom community in the investigated river sections is shown in Table 5.

The vector indicating the LP taxa shows exactly the opposite gradient as the sum of the abundances of the other algae in the phytobenthos. The gradient of SI based on other algae and the average depth of the water are also in the opposite direction than the proportion of the LP taxa, but the correlation is somewhat less clear.

The proportion of planktonic taxa increased with water depth. These species are introduced to the phytobenthos with sedimentation from the water column, which is more intense at greater depths and lower velocities, respectively.

The proportion of motile taxa increased with the temperature and depth of the water. Deeper water generally also means lower current velocities over the substrate, providing better conditions for motile species. Furthermore, deeper water also reduces light intensity, reaching the phytobenthos at the bottom. Higher temperatures also mean higher metabolic rates, which are generally higher in motile species than in other types.

## 3. Discussion

### 3.1. Differences between Natural and Regulated River Sections

The structure of the phytobenthic diatom community did not show lower diversity in regulated river reaches than in preserved natural river reaches (Table 1). On the contrary, we even found higher species richness and significantly higher (*p* = 0.02) diversity (SDI) of the diatom community in samples from regulated river reaches compared to natural ones. The effect of disturbance events on the diversity of a given community depends on its frequency and intensity, as formulated in the intermediate disturbance hypothesis [34]. It seems that in our case, a more disturbed environment allowed for greater diversity of diatoms.

Most of the environmental parameters assessed differed significantly between natural and regulated river sections (Table 2). Conductivity was significantly higher in the regulated river sections (*p* = 0.005), while channel shading was higher in the natural river sections, as expected, due to frequent removal of woody riparian vegetation along the channelized sections. However, the higher amounts of available nutrients and light in the channelized river sections had a positive effect on the benthic diatom community, resulting in higher diversity compared to the natural sections.

The diversity of the benthic diatom community was significantly higher in the regulated river sections, mostly due to the M taxa, which had significantly higher species richness (*p* = 0.018) and diversity (*p* = 0.029), as well as the proportion of M species (*p* = 0.024) in the diatom community of the regulated river sections (Table 1). The proportion of M taxa in the diatom community in the channelized rivers reached 25% (Table 1), but it accounted for nearly half of all diatom species present on average (13 out of 27 on average per sample). The motile taxa have adaptive advantages to dominate in nutrient-rich environments (e.g., nutrient storage and the ability to choose their microhabitat through motility [35]). The regulated river sections had significantly higher conductivity compared to the natural river sections (Table 2), which may be associated with higher nutrient content, which is also reflected in higher values of TI (Table 1). Channelized river sections are also more frequently exposed to strong disturbances, such as spates or extremely low water levels [36]. The motility of the M taxa enables them to find the best place in the microhabitat to avoid disturbances (i.e., to resist moderate water discharges or to reach the best position to acquire nutrients [35]). In addition, motile taxa can reach the optimal habitat (high nutrient content and moderate disturbance) faster than the other ecological types of diatoms [37,38], and they can avoid negative impacts, such as sedimentation of particles in turbid water (e.g., Figure 2).

Better adaptation of motile species to environments with disturbances and higher nutrient levels than the LP and HP taxa may explain their higher proportions and diversity in the phytobenthic diatom communities in regulated river sections. Significantly higher values of the trophic and saprobic indices in regulated river sections (Table 1), reflecting higher concentrations of nutrients and degradable organic matter, respectively, could be the consequence of higher pressure from agricultural land in lowlands, where most regulated river sections are located, combined with their lower self-purification capacity.

Taxa of the LP type are resistant to high water flow and are capable of rapid colonization of bare surfaces and substrates, while the HP taxa are more sensitive to disturbances [39]. Goldenberg Vilar et al. [40] reported that the LP taxa dominated in nutrient-poor, clear water but were absent in an enclosure with artificially low turbidity. This is due to the fact that the LP taxa also include pioneer taxa, which are the first to colonize natural habitats after severe disturbances due to their resistance to flushing. Their dominance in both natural and regulated river sections (57 and 50%, respectively) was most likely due to the mass effect (e.g., spates) of the disturbance period rather than their adaptation to the new environment.

The position of the HP taxa closer to the upper layer of the phytobenthic community enables them to have faster access to dissolved nutrients compared to other ecological types of diatoms, especially the LP taxa that are positioned in the lower layer of the periphyton [37,39]. In low nutrient and light availability, when the development of a thicker three-dimensional biofilm is prevented, the HP taxa usually dominate [41]. This explains their higher proportion (25.8%) in natural river sections, where they are more abundant than the motile taxa.

We determined significant differences between the abundances of several motile species in natural and regulated river sections (see Table 3), namely, *Nitzschia pura*, *N. fonticola*, *Surirella minuta*, and *Denticula tenuis*. The high-profile taxa *Diatoma moniliformis*, *Diatoma vulgaris*, *Didymosphenia geminate*, and *Gomphonema pumilum* were significantly more abundant on average in natural sections and may serve as indicator taxa for preserved river sections. There were also some other differences in diatom community composition between the two types of sections (Table 4), such as *Stephanocyclus meneghinianus*, *Melosira varians*, *Navicula gregaria*, and *Mayamea atomus*, which were found to be characteristic of regulated sections.

Based on our results, M diatoms can also be used to assess the ecological impact of hydromorphological pressures, as this ecological type is characteristic for channelized sections. Because this type has a significantly higher proportion (*p* = 0.024), as well as a higher number of taxa (*p* = 0.018), in channelized sections, it raises the diversity of the total benthic diatom community in channelized sections above the diversity in natural sections.

### 3.2. Influence of Selected Environmental Factors on the Structure of the Diatom Community

**Figure 3:**
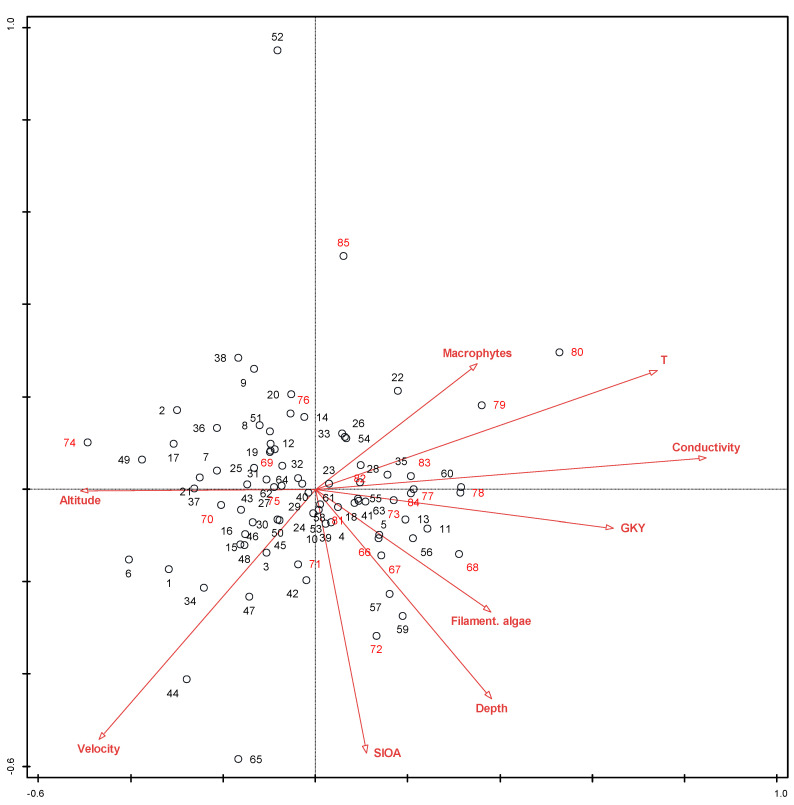
CCA ordination diagram based on the entire species matrix. Factors that significantly influenced species composition are included only. 1–65: natural river sections; 66–85: channelized river sections. Abbreviations are explained in the heading to Table 5.

**Figure 4:**
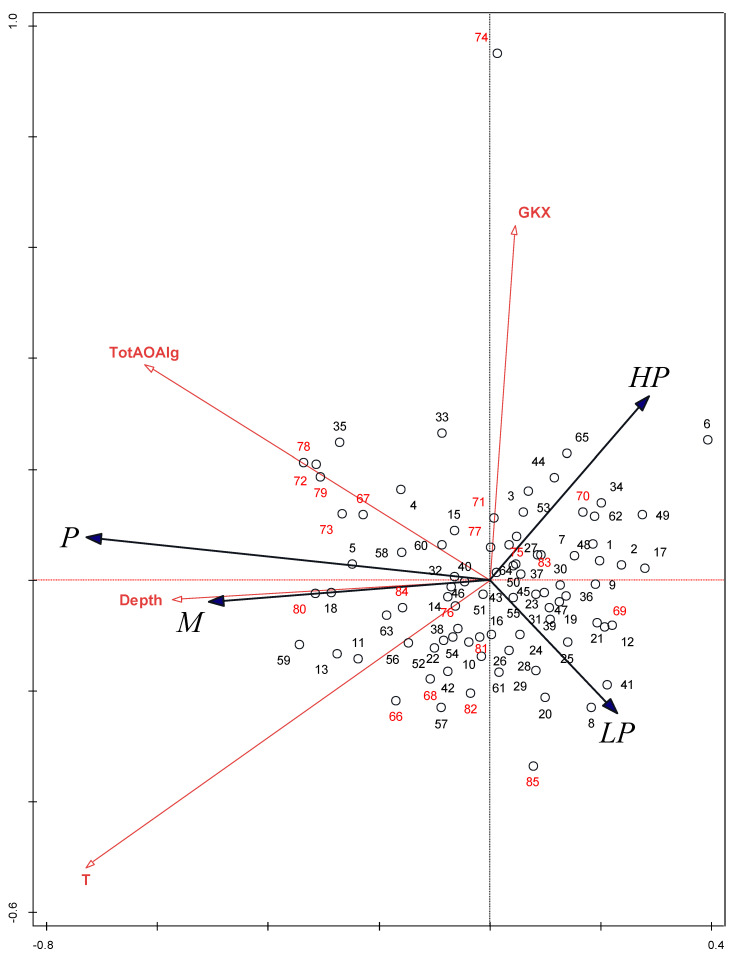
RDA ordination diagram based on the proportions of ecological types of diatoms within the phytobenthic community. Factors that significantly influenced species composition are included only. LP, low-profile diatom taxa; M, motile diatom taxa; P, planktonic diatom taxa; HP, high-profile diatom taxa. 1–65: natural sections of the rivers; 66–85: channelized sections of the rivers. Abbreviations are explained in the heading to Table 5.

**Figure 5:**
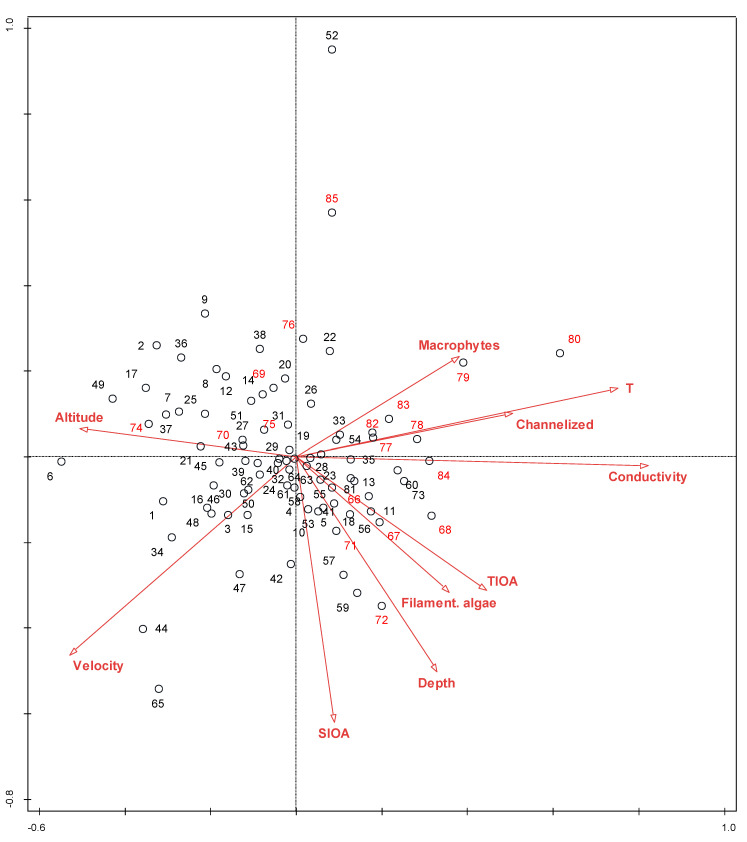
CCA ordination diagram based on the taxonomic matrix without *A. minutissimum*. Factors that significantly influenced species composition are included only. 1–65: natural river sections; 66–85: channelized river sections. Abbreviations are explained in the heading to Table 5.

The ordination diagrams show that both groups of samples originating from regulated and natural river sections overlap (e.g., Figure 3). The same result was confirmed when the species composition matrix was replaced by the ecological types matrix (Figure 4). The species matrix without *A. minutissimum* gave clearer results than the entire matrix (Figure 5), and whether the river was channelized or not became a significant factor. This indicates the irrelevance of this taxon, which is indifferent to most environmental conditions and reduces the differences and significance of various indices based on the diatom community. *A. minutissimum* is a complex of several different species that are difficult to separate; therefore, to avoid misidentification, many taxonomists prefer the *A. minutissimum* complex to the identification of individual species, regardless of the fact that species classified to the *A. minutissimum* complex have different ecological preferences. Szczepocka and Želazna-Wieczorek [42] have already reported that an excessive number of widespread generalist taxa, such as *A. minutissimum,* is the reason for inaccuracies in the assessment of ecological status. Therefore, we suggest deleting or ignoring *A. minutissimum* prior to calculating SI, TI, or other diatom-based indices when it is not determined reliably to the level of individual species, which is rare, but rather at the level of the *A. minutissimum* complex.

The proportion of variance explained by the set of parameters was much higher when the matrix of diatom species was replaced by the matrix of ecological types (Table 5), confirming the sense of such a classification in which a large number of taxa generates a complexity that is difficult to explain. This is consistent with Salmaso et al. [43], who reported that ecological types group together species with similar adaptive strategies that correspond to the actual compartments of an ecosystem to potentially simplify its complexity. This concept is promising in the case of benthic diatoms [37].

Factors that significantly influence the diatom community in both cases (taxa and ecological types) are temperature and average depth of the water. Temperature has been most frequently cited as a significant factor in shaping diatom community composition (e.g., Zelnik and Sušin [44], Toman et al. [45], Izagirre and Elosegi [25], and Soininen [46]). In the case of ecological types, the total abundance of the other algae is also a significant factor opposing the proportion of the LP taxa (Figure 4), which cannot compete for nutrients and light with more prominent green algae or cyanobacteria. The fourth significant factor influencing the diatom community is the location of the sampling site (i.e., Gauss–Krueger coordinates), which is a spatial factor. Passy [31] and Soininen [46] discovered that spatial factors can also contribute significantly to algal distribution.

Other factors that significantly shaped the composition were conductivity, SIOA, current velocity, elevation, macrophyte cover, and filamentous algae cover. Conductivity as a significant factor has been reported by several authors (e.g., Zelnik and Sušin [44], Cantonati [47], Virtanen and Soininen [46], and Toman et al. 2014 [45]). Altitude has been reported by Soininen [46], Toman et al. [45], and Beltrami et al. [48].

## 4. Materials and Methods

### 4.1. Study Area

The area of Slovenia is only approximately 20,000 km^2^, but despite this fact, its position at the junction of four biogeographical regions, Alpine, Pannonian, Dinaric, and Mediterranean [49,50], makes up for its small size in terms of biodiversity. Moreover, the geomorphological, geological, and climatic conditions in Slovenia are very diverse. As a result, great diversity is found in many biotic communities [51].

The territory of Slovenia is divided into two drainage basins: the Black Sea or Danube basin, which make up the largest part of the Slovenian territory (about 16,500 km^2^), and the Adriatic Sea basin, which covers approximately 3750 km^2^ [52]. There are four hydro-ecoregions defined in the Slovenian territory, namely the Alps, the Po lowland, the Dinaric, and the Pannonian lowland. Several rivers in the Dinaric hydro-ecoregion are intermittent, while many rivers in the Alpine ecoregion have a torrential character and cause frequent disturbances due to substantial fluctuations in water discharge [53].

**Figure 6:**
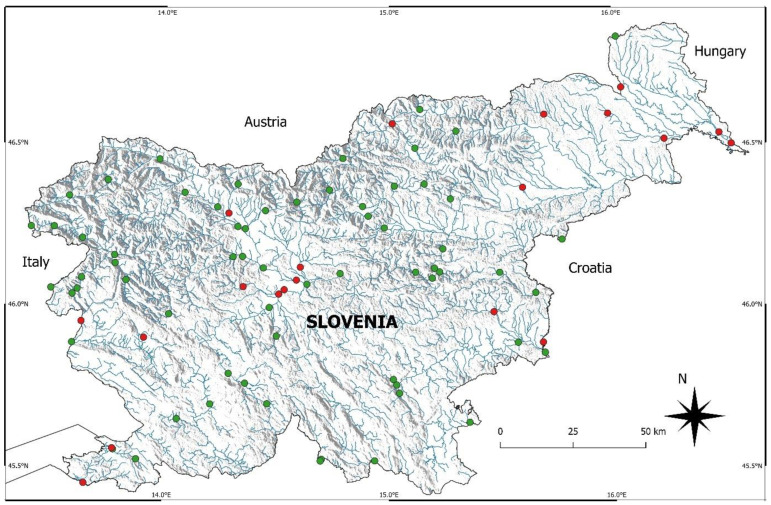
Hydrological network of Slovenia with marked sampling sites. Green circles—sampling sites in reaches with natural or non-regulated river channels; red circles—sampling sites in reaches with regulated river channels.

Samples of phytobenthos were collected in 40 rivers from both basins and all four hydro-ecoregions, namely Soča, Idrijca, Bača, Idrija, Nadiža, Vipava, Rižana, Dragonja, Reka, Pivka, Mali Obrh, Stržen, Unica, Ljubljanica, Gradaščica, Iška, Sava, Kamniška Bistrica, Kokra, Tržiška Bistrica, Sora, Savinja, Dreta, Paka, Bolska, Hudinja, Gračnica, Krka, Črmošnjica, Sotla, Bistrica, Kolpa, Meža, Mislinja, Ribnica, Ložnica, Pesnica, Ščavnica, Kučnica, and Ledava (Figure 6).

The geology of the catchment areas of the examined rivers is diverse, with a predominance of sedimentary rocks. Limestone, dolomite, sandstone, marl, and chalk–marl cover 65% of the surface of Slovenia, whereas gravel, sand, loam, clay, and other loose sediments represent an additional 29%. Metamorphic rocks cover a small proportion (4.1%), while igneous rocks are rare, covering 1.6% of the surface [54]. The climate in Slovenia is also diverse—three types are defined, including temperate humid climate, temperate continental climate, and mountain climate [55]. There is a significant west–east gradient in the precipitation amount, which is highest in the Julian Alps (NW Slovenia), where it is three times higher than the precipitation in the Pannonian lowland in the NE, where it drops to 800–900 mm per year.

### 4.2. Phytobenthos Sampling

Sampling of the phytobenthos was carried out in 2011–2014 as part of the national monitoring of surface water quality according to the WFD [22]. In total, the phytobenthos was sampled at 85 sampling sites throughout Slovenia. For the sampling sites that were analyzed more than once in the years studied, only the data from the latest sampling were included in this research.

According to the Slovenian national methodology of ecological status assessment for rivers using the phytobenthos and macrophytes [56], samples were collected in rivers up to a depth of 60 cm with at least 1 m distance from the river banks, or in cases of smaller rivers, at least 10% of the river width from the river banks. Multi-habitat sampling of the phytobenthos was performed, meaning that the samples were collected from various habitats (gravel, mud, rifle, and pool), where the stretch was heterogeneous, with different characteristics, such as substrate, depth, current velocity, and shadiness. The periphyton was removed from the substrate (stones, pebbles, wood, etc.) with a toothbrush or with a scalpel in a tray along with a small amount of river water, homogenized, and poured into a wide-necked plastic bottle. The sample was fixed with formaldehyde to a final concentration of 4%.

### 4.3. Assessment of Environmental Parameters

In accordance with the Slovenian national methodology for the assessment of the ecological status of rivers based on the phytobenthos and macrophytes [56], in addition to phytobenthos sampling, selected environmental parameters were also assessed at each sampling site, as described below. Basic physical and chemical parameters were measured in situ, namely water temperature, dissolved O_2_ concentration and saturation, conductivity, and pH using a multimeter (Eutech Instruments PCD 650, Singapore).

The water current velocities were estimated according to a 4-level scale (1: 0–10 cm/s, 2: 10–30 cm/s, 3: 30–60 cm/s, and 4: >60 cm/s), and the water depth was also estimated in four categories (1: 1–10 cm, 2: 10–30 cm, 3: 30–60 cm, and 4: >60 cm). The turbidity of the water was estimated on a 3-level scale (1: clear, 2: moderately clear, and 3: turbid). The shadiness of the channel was assessed (in %). The substrate categories, as well as their proportions of the cover (in %) of the bottom within the sampled reach, were assessed according to AQEM 2002 [57] for each sampling site (silt and clay %, sand %, gravel %, and stones %). Based on the proportions of these types of inorganic substrates, the coarseness of the substrate was also calculated, as follows: = (1 × silt and clay (%) + 2 × sand (%) + 3 × gravel (%) + 4 × stones (%)/4); the cover of the bottom with macrophytes was also estimated (in %). The month according to daylength was defined on a scale of 1–6 (6: June and July, 5: May and August, 4: April and September, etc.). The cover of the substrate with filamentous algae was estimated with a 6-level grade (1: filamentous algae not present, 6: filamentous algae totally covering the bottom). The width of the channel, width of the channel under water, coordinates in the Gauss–Krüger system (Northing as GKX and Easting as GKY), and altitude of the sampling sites were recorded as well.

### 4.4. Laboratory Analyses

Phytobenthos samples were treated with concentrated nitric acid (HNO_3_) in order to clean them of cell contents and other organic matter, according to standard EN 14,407 and the instructions of the Slovenian national methodology of ecological status assessment for rivers using the phytobenthos and macrophytes [56]. Cleaned samples were mounted in Naphrax^®^,(Brunel Microscopes, Chippenham Whiltshire, UK) a medium with a high refractive index, for permanent slide preparation. Permanent slides were examined with a light microscope (Olympus CX41 microscope, Tokyo, Japan) at a magnification of 1000×. The identification and enumeration of diatoms was performed according to standard EN 14,407 and the Slovenian national methodology of ecological status assessment for rivers using the phytobenthos and macrophytes [56]. At least 500 valves were counted and determined to the species or lower taxonomic levels for each permanent slide. For the determination and nomenclature of diatoms, the identification monographs of Kramer and Lange-Bertalot [58,59,60,61] and Hoffman et al. [62] were used.

The taxonomic composition and abundance of other algae were also analyzed. At least 50 visual fields at 400× magnification were surveyed, and all algae were counted and determined to the genus or species level. For the determination and nomenclature of other algae, the identification monographs of Susswasserflora von Mitteleuropa [63] and John et al. [64] were used. The presence of each taxon was estimated on a 5-degree scale (1: taxon solitaire, 1–10%; 2: taxon rare, 10–25%; 3: taxon moderately present, 25–75%; 4: taxon frequent, 75–90%; 5: very abundant (en masse), 90–100%).

### 4.5. Statistical Analyses

The levels of nutrient and organic enrichment of the investigated rivers were evaluated using trophic and saprobic indices, respectively. TI and SI were calculated according to Rott et al. [65]. Both indices were calculated on the basis of diatoms and other algae as well.

Diatoms were classified into four ecological types according to Rimet and Bouchez [30], namely low profile (LP), high profile (HP), motile (M), and planktonic (P), since these types can emphasize relationships with certain environmental parameters compared to species data [30].

The influence of the recorded parameters on the composition of the diatom community was tested with direct gradient analyses, which were performed with the program package CANOCO 5.0 [66]. Preliminary detrended correspondence analysis (DCA) revealed unimodal gradients in the matrix of diatom taxa according to ter Braak and Verdonschot [67], since the eigenvalue for the first axis was higher than 0.4, and the gradient length was more than 3 standard deviations. Consequently, a series of canonical correspondence analyses (CCA) were performed. The exception was the matrix with ecological types, where the eigenvalue for the first axis was 0.10, and the gradient length that was much lower than 3 standard deviations was calculated (1.02); therefore, redundancy analysis (RDA) was performed. In all cases, forward selection, with 499 permutations performed under the full model, was used to rank the relative importance of the variables and to avoid co-linearity. Only significant variables (*p* < 0.05) were considered in further analyses. CCA and RDA ordination diagrams (biplots) were created with statistically significant factors. In addition, the analysis was performed with the entire taxonomic dataset but without the dominant taxon *Achnanthidium minutissimum* (Kütz) Czarn. Such an approach has already been used by Szczepocka et al. [18]. Since this taxon was the most abundant in both the natural and regulated river sections, it can only reduce the clarity and significance of the results. Rimet and Bouchez [30] considered *A. minutissimum* to be a pioneer species indifferent to environmental factors. Urrea-Clos and Sabater [68] also reported that *A. minutissimum* occurs within the highest number of areas and thus has a very wide ecological amplitude. Szczepocka et al. [18] also pointed out that this taxon is problematic from a taxonomic point of view. Currently, the *A. minutissimum* complex includes several species, and the ecological preferences of some species have not been studied enough yet.

Differences in characteristics between the natural and regulated river sections were tested using Student’s *t*-test (MS Excel). Because diatom species of the planktonic ecological type were absent in many samples due to the natural characteristics of Slovenian rivers that are unfavorable to phytoplankton, some of the analyses were not performed specifically for the planktonic ecological type.

## 5. Conclusions

Although a higher diversity of the benthic diatom community was expected in the natural river sections, our results confirmed the opposite—there was significantly higher diversity in the channelized sections. Significantly higher conductivity and nutrient availability (trophic index), as well as higher light availability due to less shading in the channelized river sections, are factors that favor phytobenthos growth; all were more favorable in the channelized sections, enabling greater diversity of the diatom community. Motile diatoms appear to be the most responsive and best adapted to take advantage of such altered conditions, and they increase the diversity of the benthic diatom community above levels found in natural river reaches.

This suggests that diatoms can be used to assess human alterations of rivers, which are currently evaluated (according to WFD) using the benthic invertebrate community and fish community structure. The fish community and metrics calculated based on it often appear to be less efficient because the distribution of fish populations is highly dependent on the longitudinal connectivity of the river, which is often disrupted by dams. We propose that the selected diatom taxa that showed significantly different preferences for natural or channelized river reaches (Table 3 and Table 4) should also be included in the assessment of hydromorphological pressures. At the same time, we suggest excluding the taxon *Achnanthidium minutissimum* before calculating any diatom-based index, when determined as an *A. minutissimum* complex, to increase the indicative value of the phytobenthic diatom community.

Temperature and average water depth significantly shaped the structure of the diatom community when the entire species matrix was used, excluding *A. minutissimum*, or when the matrix of ecological types was used. In addition, the set of significant parameters was different when taxa were replaced by ecological types (Table 5).

## Figures and Tables

**Table 1:** The proportions of the three ecological types of benthic diatoms according to Rimet and Bouchez [30] and their contribution to the diversity of the benthic diatom community in the natural (N) and regulated (R) sections of the investigated rivers. Indices calculated on the basis of benthic diatoms: DI, diversity index; SI, saprobic index; TI, trophic index; EQR, ecological quality ratio (blue color: very good status, green color: good status). Bold numbers represent significantly higher average values between compared groups.

Ecological Type		N		R
	Proportion [%]	Average	*p*-Value	Average
Low profile		57.3	0.199	49.6
High profile		25.8	0.063	19.5
Motile		15.0	0.024	**25.1**
	Number of diatom taxa			
Low profile		7	0.187	7.5
High profile		8	0.167	7
Motile		9.3	0.018	**13**
	Shannon–Wiener DI			
Low profile		1.07	0.026	**1.26**
High profile		1.36	0.634	1.29
Motile		1.53	0.029	**1.88**
All taxa	Number of diatom taxa	24.9	0.071	28.6
All taxa	Shannon–Wiener DI	2.02	0.020	**2.33**
All taxa	SI	1.76	0.012	**1.97**
All taxa	TI	2.06	0.002	**2.57**
All taxa	SI EQR	**1.19**	0.037	0.90
All taxa	TI EQR	**1.07**	0.003	0.77

**Table 2:** The average values of selected environmental parameters and results of Student’s *t*-test between natural (N) and regulated (R) sections of the rivers. (n.s.: non-significant).

Environmental Parameter	N		R
	Average	*p*-Value	Average
Altitude [m a.s.l.]	311.95	0.002	213.65
Temperature [°C]	16.40	n.s.	18.09
Conductivity [µS/cm]	377.04	0.005	492.40
Width of the channel [m]	56.14	0.030	33.85
Width of the channel under water [m]	40.17	0.043	22.15
Current velocity (4-level scale)	1.93	0.017	1.61
Turbidity (3-level scale)	1.2	n.s.	1.5
Sand proportion [%]	21.46	0.045	34.50
Gravel proportion [%]	55.08	0.003	36.25
Substrate coarseness (5-level scale)	3.01	n.s.	2.73
Shadiness of the channel [%]	15.77	n.s.	8.00
Filamentous algae cover [6-level scale]	4.00	n.s.	4.55

**Table 3:** Average proportion values (%) of diatom taxa were statistically significantly different between natural (N) and regulated (R) river sections. LP, low profile; HP, high profile; M, motile. Taxa highlighted in green are characteristic of N.

Ecological Type	Name	N	*t*-Test	R
		Average	*p*-Value	Average
LP	*Achnanthidium pyrenaicum* (Hustedt) Kobayasi	19.16	0.042	8.66
LP	*Planothidium dubium* (Grunow) Round and Bukhtiyarova	0.33	0.037	1.71
LP	*Cymbella affinis* Kützing	0.71	0.049	0.19
HP	*Diatoma moniliformis* (Kützing) Williams	0.11	0.045	0.00
HP	*Diatoma vulgaris* Bory	5.55	0.006	1.17
HP	*Didymosphenia geminata* (Lyngbye) Schmidt	0.10	0.017	0.00
HP	*Gomphonema pumilum* (Grunow) Reichardt and Lange-Bertalot	3.05	0.037	0.95
M	*Denticula tenuis* Kützing	1.90	0.002	0.05
M	*Nitzschia fonticola* Grunow	2.22	0.042	0.82
M	*Nitzschia pura* Hustedt	0.25	0.024	0.00
M	*Surirella minuta* Brébisson ex Kützing	0.02	0.026	0.09

**Table 4:** Average proportion values (%) of diatom taxa and the order of average proportions in natural (N) and regulated (R) river sections. LP, low profile; HP, high profile; M, motile; P, planktonic. Taxa highlighted in green are characteristic of N, while those highlighted in red are characteristic of R river sections.

Ecol. Type	Name	Order of Frequency	Average Proportions (%)		Order of Frequency
		N	N	R	R
LP	*Achnanthidium minutissimum* (Kütz.) Czarn.	1	21.9	15.2	1
LP	*Achnanthidium pyrenaicum* (Hustedt) Kobayasi	2	19.2	8.7	3
LP	*Cocconeis* cf. *placentula* Ehrenberg	3	6.3	11.3	2
HP	*Diatoma vulgaris* Bory	4	5.5	1.2	23
HP	*Melosira varians* Agardh	13	1.6	5.4	4
LP	*Cocconeis pediculus* Ehrenberg	7	2.4	4.3	5
P	*Stephanocyclus meneghinianus* (Kützing) Kulikovskiy, Genkal, and Kociolek		0.9	3.8	6
HP	*Gomphonema pumilum* (Grunow) Reichardt and Lange-Bertalot	5	3.1	0.9	
HP	*Encyonema minutum* (Hilse in Rabh.) D.G. Mann	6	2.9	2.5	10
M	*Navicula gregaria* Donkin		0.7	2.8	7
M	*Nitzschia palea* (Kützing) W.Smith *var. debilis* (Kützing) Grunow	15	1.5	2.5	8
M	*Nitzschia homburgiensis* Lange-Bertalot	20	1.0	2.5	9
HP	*Fragilaria capucina* Desmazières	8	2.3	1.0	
M	*Nitzschia fonticola* Grunow	9	2.2	0.8	
LP	*Cymbella microcephala* Grunow		0.7	2.2	11
M	*Mayamaea atomus* (Kützing) Lange-Bertalot		0.6	2.1	12
M	*Nitzschia paleacea* (Grunow) Grunow		0.3	2.0	13
HP	*Gomphonema minutum* (Agardh) Agardh	10	1.9	1.6	17
M	*Denticula tenuis* Kützing	11	1.9	0.0	
LP	*Amphora indistincta* Levkov	12	1.9	1.5	19
P	*Nitzschia acicularis* (Kützing) W. Smith		0.9	1.8	14
HP	*Gomphonema exilissimum* (Grunow) Lange-Bertalot and Reichardt		0.5	1.8	15
LP	*Planothidium dubium* (Grunow) Round and Bukhtiyarova		0.3	1.7	16
M	*Navicula tripunctata* (O.F. Müller) Bory	19	1.0	1.6	18
HP	*Encyonema silesiacum* (Bleisch) D.G. Mann	14	1.6	0.6	
M	*Nitzschia dissipata* (Kützing) *Grunow* ssp. *dissipata*		0.9	1.5	20
HP	*Gomphonema cymbelliclinum* Reichardt and Lange-Bertalot	16	1.5	0.3	
LP	*Achnanthidium saprophilum* (Kobayasi and Mayama) Round and Bukhtiyarova	17	1.5	0.7	
LP	*Rhoicosphenia abbreviata* (Agardh) Lange-Bertalot		0.8	1.4	21
HP	*Ulnaria ulna* (Nitzsch) Compere	18	1.4	0.8	
M	*Navicula menisculus* Schumann	21	1.0	1.2	22

**Table 5:** Results of canonical correspondence analyses (CCA) based on the entire species matrix and the matrix without *A. minutissimum*. Species data were log 10 (x + 1) transformed. Eigenvalues: Axis 1: 0.2718; Axis 2: 0.2020. Result of redundancy analysis (RDA) based on the proportions of ecological types. Eigenvalues: Axis 1: 0.3058; Axis 2: 0.0280. Factors that significantly influenced the species composition are listed only (*p* < 0.05). (SI, saprobic index; TI, trophic index; OA, other algae; D, diatoms).

	Entire Species Matrix, CCA		Species Matrix Without *A. minutissimum*, CCA		Matrix of Ecological Types, RDA	
Variable	Explains %	*p*	Explains %	*p*	Explains %	*p*
Conductivity	4.8	0.002	4.7	0.002		
SI OA	3.0	0.002	3.0	0.002		
Temperature	2.7	0.002	2.6	0.002	17.1	0.002
Average current velocity	2.4	0.002	2.4	0.002		
Altitude	2.4	0.002	2.4	0.002		
Macrophyte cover	2.0	0.006	2.0	0.008		
Filamentous algae cover	1.8	0.006	1.7	0.002		
GKY	1.8	0.032				
Average water depth	1.7	0.002	1.6	0.004	5.0	0.002
TI OA			2.0	0.002		
Sum abundances of other algae					8.8	0.002
GKX					3.5	0.01
Regulated/natural			1.7	0.008		
Total	22.6		24.1		34.4	

## Data Availability

Data can be provided upon reasonable request.
